# XBB.1.5 monovalent vaccine induces lasting cross-reactive responses to SARS-CoV-2 variants such as HV.1 and JN.1, as well as SARS-CoV-1, but elicits limited XBB.1.5 specific antibodies

**DOI:** 10.1128/mbio.03607-24

**Published:** 2025-03-05

**Authors:** Juan Manuel Carreño, Brian Lerman, Gagandeep Singh, Anass Abbad, Temima Yellin, Jordan Ehrenhaus, Miriam Fried, Jessica R. Nardulli, Hyun Min Kang, Lubbertus C. F. Mulder, Charles Gleason, Komal Srivastava, PVI study group, Viviana Simon, Florian Krammer

**Affiliations:** 1 Department of Microbiology, Icahn School of Medicine at Mount Sinai, New York, USA; 2 Center for Vaccine Research and Pandemic Preparedness (C-VaRPP), Icahn School of Medicine at Mount Sinai, New York, USA; 3 The Global Health and Emerging Pathogens Institute, Icahn School of Medicine at Mount Sinai, New York, USA; 4 Department of Pathology, Molecular and Cell Based Medicine, Icahn School of Medicine at Mount Sinai, New York, USA; 5 Division of Infectious Diseases, Department of Medicine, Icahn School of Medicine at Mount Sinai, New York, USA; 6 Ignaz Semmelweis Institute, Interuniversity Institute for Infection Research, Medical University of Vienna, Vienna, Austria; McMaster University, Hamilton, Ontario, Canada

**Keywords:** SARS-CoV-2, cross-reactive immune responses, COVID-19 vaccine, XBB.1.5 monovalent vaccine, imprinting

## Abstract

The evolution of the antibody response to the severe acute respiratory syndrome coronavirus 2 (SARS-CoV-2) is impacted by the nature and number of antigenic exposures. First-generation coronavirus disease 2019 (COVID-19) vaccines encoded an ancestral spike protein. Updated bivalent vaccines and breakthrough infections have shaped the intricate diversity of the polyclonal antibody response and specificity of individual antibody clones. We and others previously showed that bivalent vaccines containing the ancestral and Omicron (BA.5) spikes induce high levels of cross-reactive antibodies but undetectable BA.5-specific antibodies in serum. Here, we assessed sera collected before as well as 1 and 3 months following administration of an updated XBB.1.5 monovalent vaccine to individuals with diverse infection and vaccination histories. Vaccination increased neutralization against recent variants of concern, including HV.1, JN.1, and the vaccine-homologous XBB.1.5. Antibody binding and avidity against ancestral and XBB.1.5 antigens significantly increased after vaccination. However, antibody depletion experiments showed that most of the response was cross-reactive to the ancestral spike, and only low levels of XBB.1.5-specific antibodies to the spike or the receptor-binding domain were detected. Importantly, increased antibody levels were still detectable in circulation 3 months post-vaccination and cross-reacted with severe acute respiratory syndrome coronavirus 1 (SARS-CoV-1) as measured by pseudovirus neutralization and binding assays. Overall, our data suggest that the XBB.1.5 monovalent vaccine predominantly elicits a cross-reactive response imprinted by viral spike antigens encountered early during the pandemic.

**IMPORTANCE:**

Updated COVID-19 vaccine formulations and SARS-CoV-2 exposure history affect the antibody response to SARS-CoV-2. High titers of antibodies are induced in serum by XBB.1.5 monovalent vaccination. Antibody depletion experiments reveal that the majority of the antibody response is cross-reactive to the ancestral spike, despite vaccination increasing neutralization against recently circulating Omicron variants. Vaccine-induced SARS-CoV-2 antibodies cross-react with SARS-CoV-1 and remain in the bloodstream for at least 3 months after immunization.

## INTRODUCTION

With more than 700,000,000 documented cases and more than 7,000,000 official deaths (1) (estimates based on excess mortality exceed 20 million deaths), the coronavirus disease 2019 (COVID-19) pandemic has affected the human population in an unprecedented manner. Vaccination played a pivotal role in the containment of the virus and ameliorated the prognosis of infected individuals by reducing disease severity and, consequently, the number of hospitalizations and deaths (2, 3). Although first-generation COVID-19 vaccines encoding the Wuhan-1 or ancestral spike protein sequence were highly effective against initial viral variants including B.1.1.7 (Alpha), B.1.351 (Beta), B.1.427/429 (Epsilon), B.1.525 (Eta), P.1 (Gamma), B.1.526 (Iota), B.1.617.1 (Kappa), and C.37 (Lambda), among others (4, 5), the emergence of variants of concern from the Omicron lineage (BA.1.1.529) substantially hampered the neutralization capacity of vaccine-derived antibodies (6, 7). This, in turn, prompted vaccine updates to incorporate spike protein sequences from more recent circulating variants. From bivalent vaccines based on ancestral/BA.5 spikes to more recent monovalent vaccines incorporating XBB.1.5 spikes (8), updated vaccines aim to improve immunity to highly drifted severe acute respiratory syndrome coronavirus 2 (SARS-CoV-2) variants.

Since their emergence, SARS-CoV-2 variants increased the magnitude and diversity of the polyclonal antibody response elicited by infection with the ancestral virus and/or by administration of COVID-19 vaccines based on ancestral spike proteins (9). However, most of these antigenic exposures have primarily boosted cross-reactive responses, rather than *de novo* variant-specific antibodies (10). We and others have shown that ancestral/BA.5 bivalent vaccines elicit cross-reactive antibodies to the ancestral spike but do not induce detectable responses specific to Omicron antigens as measured by conventional serological assays (11, 12). At the monoclonal level, following administration of a monovalent BA.1 or a bivalent B.1.351 and B.1.617.2 (Beta/Delta) vaccines, *de novo* B cells targeting variant-specific epitopes can be detected, albeit at very low levels compared to cross-reactive B-cells (13).

Continuous exposures to Omicron antigens, distant from ancestral SARS-CoV-2 variants, were expected to partially overcome imprinting by ancestral antigens and redirect the antibody response to Omicron-specific epitopes (14). Specifically, breakthrough infections with highly drifted variants, including XBB.1.5 and JN.1, and administration of updated monovalent XBB.1.5 vaccines may help to redirect the antibody response to variant-specific epitopes. Here, we evaluated how individuals with complex immune histories derived from various antigenic exposures to SARS-CoV-2 infection and COVID-19 vaccination respond to the XBB.1.5 monovalent vaccine. We assessed the antibody response up to 3 months after vaccination, and our results integrated binding, avidity, and neutralization data. We found that adults receiving the XBB.1.5 vaccine mounted robust antibody responses that were maintained over time. These antibodies were mostly cross-reactive in nature, neutralizing recent variants of concern, including XBB.1.5, HV.1, and JN.1, as well as the beta coronavirus severe acute respiratory syndrome coronavirus 1 (SARS-CoV-1). Finally, vaccination-derived antibodies displayed high avidity, but only few individuals produced low levels of XBB.1.5-specific antibodies.

## RESULTS

### XBB.1.5 monovalent vaccination induces lasting neutralizing serum antibodies against WA.1, HV.1, JN.1, and homologous XBB.1.5 virus

**Fig 1:**
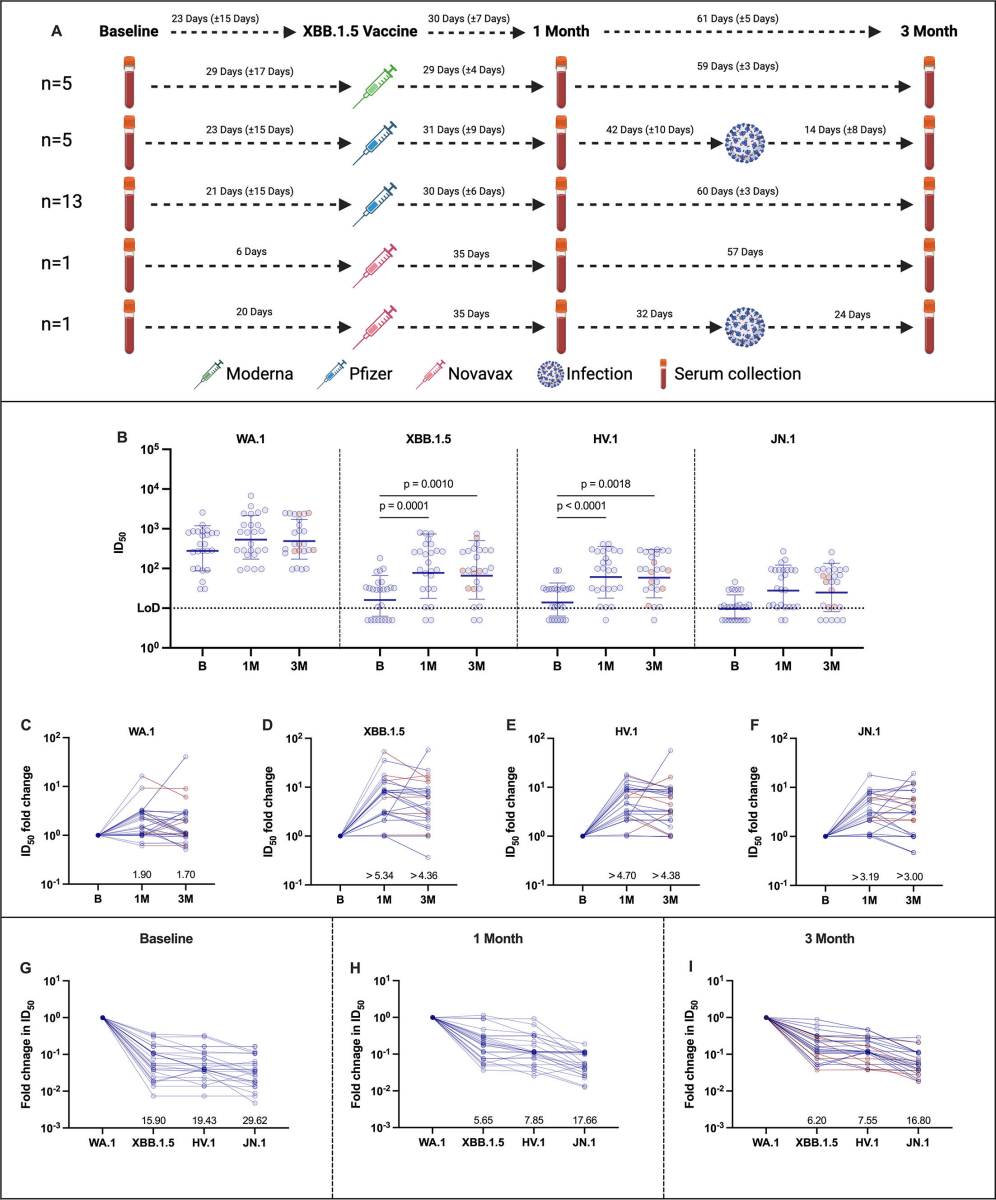
Serum neutralization profile against ancestral WA.1, XBB.1.5, HV.1, and JN.1 in individuals with diverse exposure histories receiving an XBB.1.5 monovalent vaccine. Schematic representation of recent immune history and sample collection time points in study participants (**A**). Live virus neutralization titers expressed as 50% inhibitory dilution (ID_50_) at baseline and 1 or 3 months post-vaccination against ancestral WA.1, XBB.1.5, HV.1, and JN.1 (**B**). Fold change (increase) in ID_50_ values at 1 and 3 months post-vaccination with respect to baseline reactivity (**C–F**). Fold difference (decrease) in ID_50_ values for XBB.1.5, HV.1, and JN.1 with respect to ancestral WA.1 (**G–I**). In panel **A**, individuals were stratified based on each vaccine type and presence/absence of a breakthrough infection prior to the 3 month serum collection time point. The sample size for each group is indicated on the left side. The timing (average number of days ± standard deviation) between each event is indicated above every dashed arrow. Further details are provided in Table S2; and Fig. S1. In panel **B**, Friedman’s test, followed by Dunn’s multiple comparison test, was used among different groups. Only statistically significant differences are shown. Bars = geometric means; error bars = geometric standard deviations. The assay limit of detection (LoD) is indicated by the horizontal dotted line. Values at the LoD are positive for neutralization at a 1:10 dilution, while values below the LoD are indicated as half of the LoD for graphing purposes. In panels **B–I**, individuals with a breakthrough infection between 1 and 3 months post-vaccination (*n* = 6) are highlighted in red. Each symbol represents a single participant. In panels** C–I**, the average fold change is indicated above the *x* axis.

To assess the antibody responses elicited by the XBB.1.5 monovalent vaccine, we collected samples from 25 adults enrolled in observational longitudinal studies at the Icahn School of Medicine at Mount Sinai (see Materials and Methods for details; Fig. 1) (15). Participants included in the study received either a Moderna (Spikevax, *n* = 5), Pfizer (Comirnaty, *n* = 18), or Novavax (*n* = 2) XBB.1.5 monovalent vaccine and had a different number of prior antigenic exposures (Fig. S1A). Sera were collected prior to administration of the vaccine and, on average, at 1 and 3 months following vaccination. Six individuals, five in the Pfizer group and one in the Novavax group, had a breakthrough infection between the 1 month (second) and 3 month (third) blood draw. Overall, the study population was representative of the general population that has undergone a variable number of infections and vaccinations (Fig. S1B through D).

We measured neutralizing antibody titers against WA.1, XBB.1.5, HV.1, and JN.1 using a replication-competent SARS-CoV-2-based neutralization assay (Fig. 1B). A significant increase in titers was detected against all viruses 1 month post-vaccination, which was maintained at the 3 month time point, except for the ancestral virus (wild type [WT], here USA-WA.1/2020 [WA.1]). Moreover, substantial antibody fold induction was detected against all the SARS-CoV-2 isolates tested; this increase was maintained at 3 month post-vaccination (Fig. 1C through F). Baseline antibody levels (prior to XBB.1.5 vaccination) against XBB.1.5, HV.1, and JN.1 displayed the following neutralization pattern with respect to the ancestral virus: WA.1 (ancestral) > XBB.1.5 > HV.1 > JN.1. These data are in good agreement with the findings reported by Wang and colleagues (16) (Fig. 1G through I). This pattern was consistent at 1 and 3 months post-vaccination, albeit at a higher magnitude as compared to baseline. Of note, neutralizing antibody levels in the six participants with a documented breakthrough infection after XBB.1.5 vaccination—highlighted in red in all the figures—were scattered among the values of the individuals without breakthrough infections.

### Vaccination induces predominantly a cross-reactive antibody response and low XBB.1.5-specific antibody levels

**Fig 2:**
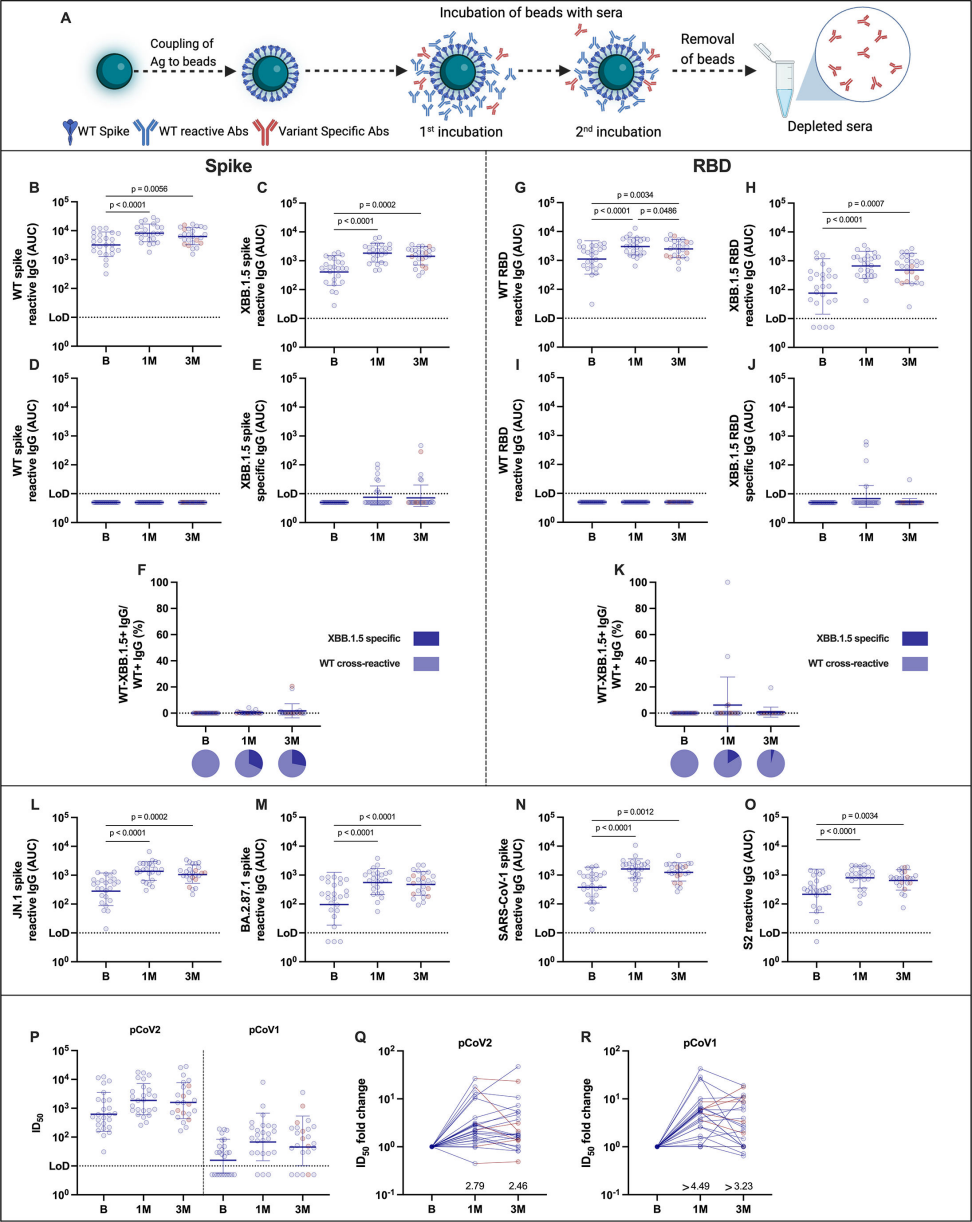
Binding and cross-reactivity profiles of sera from XBB.1.5 vaccine recipients. Schematic representation of antibody depletion used to characterize XBB.1.5 binding-specific antibodies (**A**). Briefly, chemically activated magnetic beads are coupled with recombinant spike or receptor-binding domain (RBD) proteins, followed by incubation with the target serum sample and separation of non-bound antibodies after two rounds of depletion. Binding antibody levels expressed as area under the curve (AUC) at baseline, 1 or 3 months post-vaccination against recombinant ancestral Wuhan-1 or XBB.1.5 spike (**B and C**) and RBD (**G and H**). Confirmation of antibody depletion using ancestral Wuhan-1 spike or RBD (**D and I**). Wuhan-1 spike or RBD depleted sera assessed against XBB.1.5 spike or RBD (**E and J**). Proportion of XBB.1.5-specific or Wuhan-1 cross-reactive, spike, or RBD antibodies (**F and K**). Values exceeding 100% are shown as 100%. Pie charts show the proportion of individuals who have any XBB.1.5-specific responses. Cross-reactive binding antibodies against the spike of JN.1 (**L**), BA.2.87.1 (**M**), and SARS-CoV-1 (**N**), or against the S2 domain of SARS-CoV-2 (**O**). Cross-reactive neutralizing antibodies expressed as 50% inhibitory dilution (ID_50_) measured in a single cycle pseudovirus neutralization assay against SARS-CoV-1 (pCoV1, right panel) (**P**). SARS-CoV-2 D614G pseudovirus was included as reference (pCoV2, left panel). Fold change (increase) in ID_50_ values at 1 and 3 months post-vaccination with respect to baseline titers in SARS-CoV-2 (**Q**) and SARS-CoV-1 (**R**) pseudovirus neutralization assays. In panels **B–O**, Friedman’s test, followed by Dunn’s multiple comparison test, was used among different groups. Only statistically significant differences are shown. Bars = geometric means; error bars = geometric standard deviations. The assay limit of detection (LoD) is indicated by the horizontal dotted line. Values at the LoD are positive for binding or neutralization at a 1:10 dilution, while values below the LoD are indicated as half of the LoD for graphing purposes. In panels **B–Q**, individuals with a breakthrough infection between 1 and 3 months post-vaccination (*n* = 6) are highlighted in red. Each symbol represents a single participant. In panels **P–Q**, the average fold change is indicated above the *x* axis. The ID_50_ values calculated for the pseudovirus-based neutralization assays represent the titer (dilution) at >50% inhibition. Reactive antibodies are antibodies directed to the spike/RBD of a particular strain. Specific antibodies are antibodies directed specifically to the spike/RBD of a particular strain with no reactivity to the antigens of the other strains tested.

Previously, we showed that bivalent vaccines incorporating BA.5 and ancestral spike protein sequences induced a cross-reactive response rather than variant-specific antibodies. To assess if the XBB.1.5 monovalent vaccination induces XBB.1.5-specific antibodies, we performed antibody depletion experiments and assessed reactivity by an enzyme-linked immunosorbent assay (ELISA). Briefly, the antigen for depletion is incubated with magnetic beads, followed incubation of the antigen-coated beads with the serum sample to be depleted, and finally, recovery of depleted sera by magnetic separation of the beads (Fig. 2A). Like neutralizing antibodies, binding antibodies to ancestral or XBB.1.5 antigens, including spike (Fig. 2B and C) and receptor-binding domain (RBD) (Fig. 2G and H), increased after vaccination and were maintained at the 3 month time point. Antibody depletion was performed using ancestral spike or RBD and was confirmed by ELISA (Fig. 2D and I). XBB.1.5 spike- and RBD-specific antibodies were detected in a fraction of individuals after vaccination, albeit at low levels, typically below 1,000 area under the curve (AUCs) values (Fig. 2E and J). Of all participants, 32% had detectable XBB.1.5-specific spike antibodies 1 month and 28% at 3 months post-vaccination (Fig. 2F), while these proportions were 16% and 4% for XBB.1.5-specific RBD antibodies, respectively (Fig. 2K). Thus, the XBB.1.5 monovalent vaccine mostly induces a cross-reactive antibody response, with limited induction of XBB.1.5-specific antibodies.

Next, we characterized the cross-reactivity of the polyclonal antibody responses before and after XBB.1.5 vaccination. In line with neutralization data, vaccination boosted cross-reactive binding antibodies against JN.1, BA.2.87.1, and SARS-CoV-1 spikes, and the S2 domain of SARS-CoV-2 (Fig. 2L through O). To assess if this profile encompassed neutralization of SARS-CoV-1, we used a single-cycle pseudovirus-based SARS-CoV-1 assay (17, 18) in which the luciferase read-out represents the quantity of pseudovirus entry into the target cells. Briefly, virus-like particles (VLPs) were generated by co-transfecting a lentiviral vector expressing luciferase with codon-optimized spike proteins from SARS-CoV-1 or ancestral SARS-CoV-2 (G614, control). Titered SARS-CoV-1 or SARS-CoV-2 pseudotyped VLPs were incubated with serially diluted plasma prior to addition to target cells. Two days later, luciferase activity was quantified in cell lysates to provide read-outs for cell entry. Vaccination increased the inhibition of not only ancestral SARS-CoV-2 pseudotyped VLP but also SARS-CoV-1 pseudotyped VLPs substantially (Fig. 2P through R). Of note 10 of 25 (40%) participants had no detectable neutralizing SARS-CoV-1 antibodies prior to vaccination, but 1 and 3 months after vaccination, the number of participants with SARS-CoV-1 neutralization titers below detection decreased to three and five, respectively. These neutralization titers were maintained over time (Fig. 2P). Fold induction at 1 or 3 months post-vaccination was comparable for SARS-CoV-1 and SARS-CoV-2 (from different baseline levels), although the fold increase for SARS-CoV-1 neutralization cannot be calculated accurately, given that 40% of the samples prior to vaccination had no detectable neutralization activity (Fig. 2Q). Taken together, these pseudovirus-based coronavirus neutralization data provide additional evidence that the antibody response is predominantly cross-reactive.

### Increases in antibody binding and neutralization correlate with higher antibody avidity

**Fig 3:**
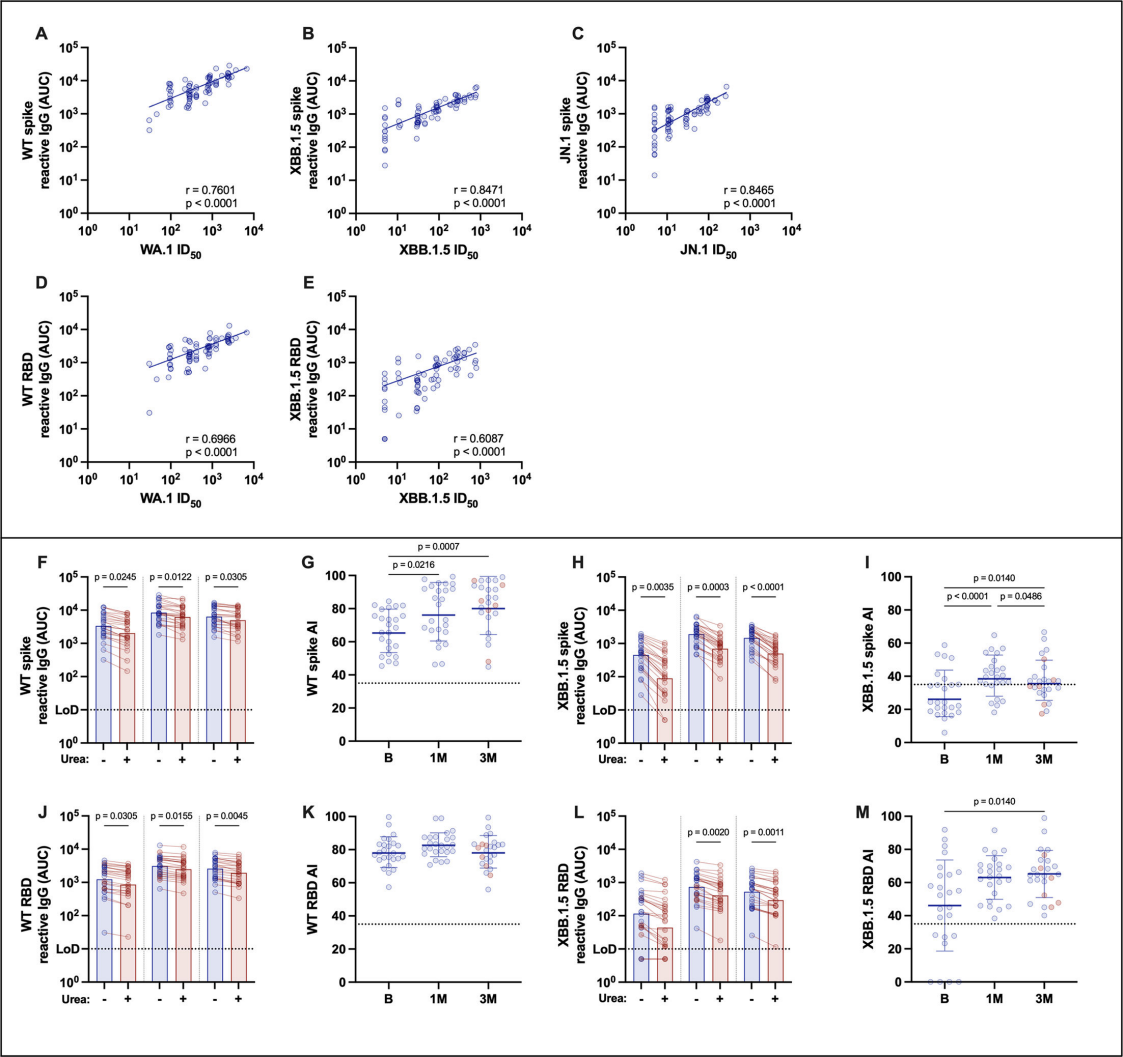
Antibody avidity profile in sera from XBB.1.5 vaccine recipients. Correlation between binding antibody levels against WT, XBB.1.5, or JN.1, spike or against the receptor-binding domain (RBD) of ancestral and XBB.1.5 viruses, and neutralization against ancestral WA.1, XBB.1.5, or JN.1 viruses (**A–E**). Antibody avidity was measured by ELISA using 8 M urea. Reactivity against the indicated antigens of plates treated with urea (red bars) or not treated (blue bars) is shown. Ancestral spike (**F**), XBB.1.5 spike (**H**), WT RBD (**J**), and XBB.1.5 RBD (**L**). Avidity index [(urea-treated sample AUC/non-treated sample AUC) × 100] of samples against ancestral spike (**G**), XBB.1.5 spike (**I**), ancestral RBD (**K**), and XBB.1.5 RBD (**M**) are shown. Friedman’s test, followed by Dunn’s multiple comparison test, was used among different groups. Only statistically significant differences are shown. Bars represent the geometric means, with error bars depicting the geometric standard deviations. Correlation coefficient (*R*) and significance value (*P*) are indicated above the *x* axis. AUC, area under the curve; ID_50_, 50% inhibitory dilution.

Levels of SARS-CoV-2 spike-binding antibodies correlate with the magnitude of the neutralizing antibody response (19, 20). We assessed how these features correlated after administration of the XBB.1.5 monovalent vaccine: we detected a strong correlation between the binding levels of antibodies reactive to the WT, XBB.1.5, or JN.1 spikes and neutralization of these viruses (Fig. 3A through C). Likewise, we noted a good correlation between WT- or XBB.1.5 RBD-binding antibodies and neutralization of these viruses, albeit this was lower for XBB.1.5 RBD (Fig. 3D and E).

Given the strong correlation detected between binding and neutralizing antibodies, we decided to measure antibody avidity in XBB.1.5 vaccine recipients. Antibody avidity is impacted by the affinity of individual antibody clones and informs about the strength of interactions between polyclonal antibodies and their epitopes. To assess antibody avidity, we used a chaotropic agent added after incubation of serial dilutions of polyclonal serum with the antigen (19). Overall, we detected high antibody avidity against ancestral spike (Fig. 3F and G) and RBD (Fig. 3J and K) proteins at baseline, with a modest increase at 1 month after vaccination. These levels seemed to remain steady at the 3-month time point. Avidity against XBB.1.5 spike (Fig. 3H and I) and RBD (Fig. 3L and M) was lower at baseline in most individuals (<35 avidity index [AI]), increased 1 month after vaccination, and remained steady at 3 months. The average AIs (ratio of binding antibodies under urea treatment relative to an untreated sample) started at 65.3 and 78.0 for WT spike and RBD, respectively. For spike, the AIs increased to 76 (1 month) and 80 (3 month), while the AIs for RBD were 82 (1 month) and 78 (3 month) for RBD. For XBB.1.5 antigens, the AIs started at 26 and 51 for spike and RBD, respectively, and increased to 38 (1 month) and 36 (3 month) for spike and to 62 (1 month) and 64 (3 month) for RBD.

### XBB1.5 vaccination induces a poor IgM response of cross-reactive nature

**Fig 4:**
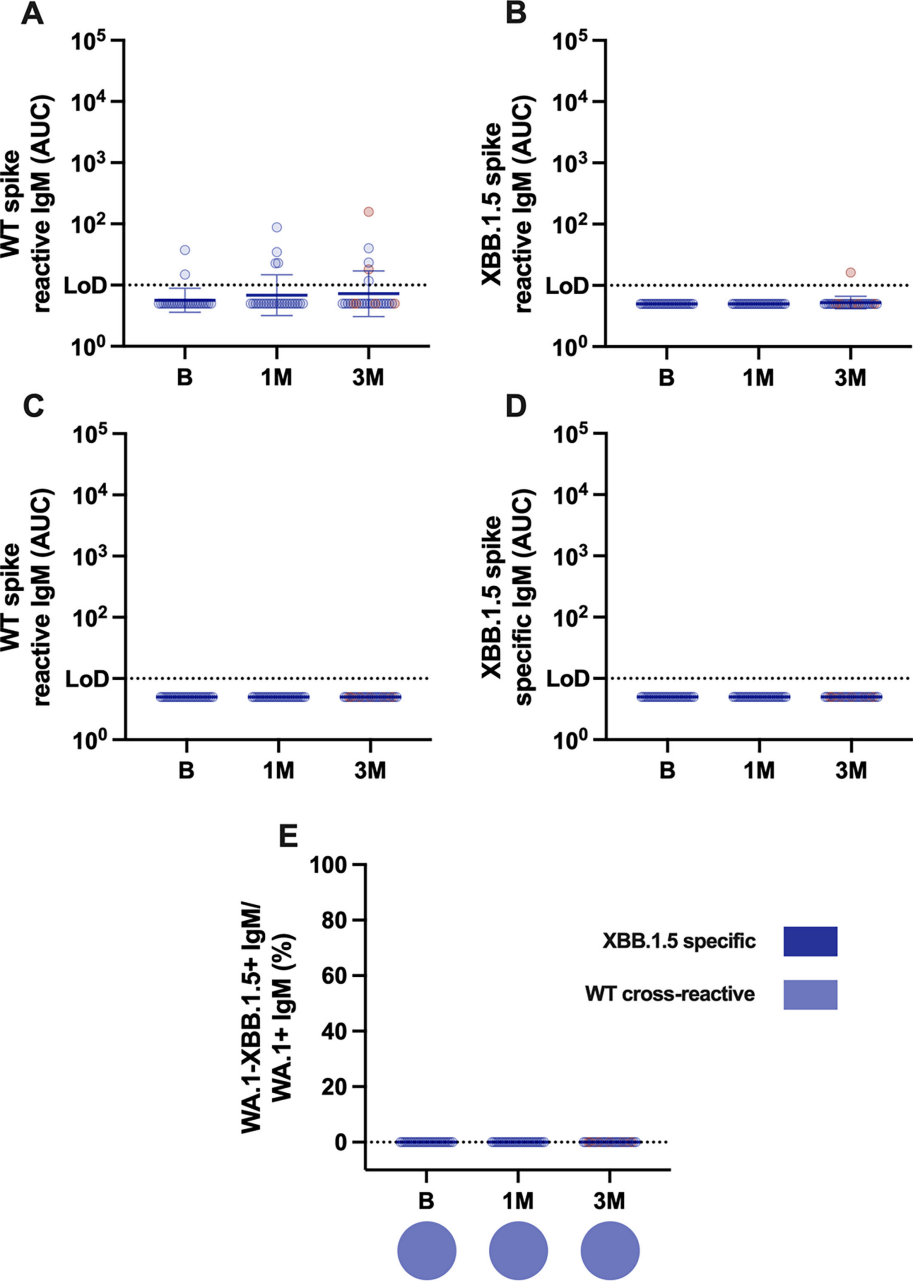
IgM measurement in sera from XBB.1.5 vaccine recipients. IgM antibody levels measured at baseline and 1 or 3 months post-vaccination against recombinant ancestral Wuhan-1 or XBB.1.5 spike (**A and B**). Confirmation of antibody depletion using ancestral Wuhan-1 spike (**C**). Wuhan-1 spike depleted sera assessed against XBB.1.5 spike (**D**). Proportion of XBB.1.5 spike-specific or Wuhan-1 spike cross-reactive antibodies (**E**). Pie charts show the proportion of individuals with any specific responses to XBB.1.5 spike. An analysis of variance test was used for statistical comparisons among different groups. Only statistically significant differences are shown. Bars = geometric means; error bars = geometric standard deviations. The assay limit of detection (LoD) is indicated by the horizontal dotted line. Values at the LoD indicate positive binding at a 1:10 dilution, while values below the LoD are shown as half of the LoD for graphing purposes.

To analyze if the monovalent XBB.1.5 vaccine induced *de novo* IgM antibodies specific to the XBB.1.5 spike protein, we performed antibody depletion experiments and measured spike-specific IgM. In general, spike IgM levels were low and detected only in a few individuals (5 of 25 with two vaccinees having low but detectable IgM prior to vaccination). Moreover, we found no significant differences in spike IgM levels at 1 and 3 months post-vaccination (Fig. 4A). IgM antibodies were specific for ancestral spike and were not detected against XBB.1.5 spike (Fig. 4B). This was confirmed by depletion of ancestral spike reactive antibodies, which removed all detectable antibodies against this antigen (Fig. 4C), but antibodies specific for XBB.1.5 spike were also not detected under these experimental conditions (Fig. 4D and E).

## DISCUSSION

The main concern about the evolutionary pathway of SARS-CoV-2 is the emergence of viral variants capable of overcoming population immunity derived from prior infection and vaccination. Indeed, recent highly drifted viral strains are poorly neutralized by polyclonal sera from recipients of ancestral Wuhan-1 mRNA vaccines or sera from those previously infected with ancestral SARS-CoV-2 variants (6, 7). As consequence, updated vaccines incorporating the Wuhan-1 spike and the spike from variants of the Omicron lineage, namely, bivalent vaccines, were initially implemented (21). However, these vaccines turned out to be insufficient to overcome imprinted responses to antigens from initial exposures and induced only cross-reactive responses to the Wuhan-1 spike (22). Recently, monovalent vaccines with updated spike antigens from Omicron XBB.1.5 were approved, requiring the characterization of immune responses to these novel vaccines.

Essential questions include (i) whether monovalent Omicron-specific vaccines will be able to overcome the responses imprinted by ancestral Wuhan-1 antigens; (ii) what the longevity of these responses is; and (iii) what the pattern of neutralization of sera from XBB.1.5 vaccine recipients is against the drifted, recent variants of concern. By performing depletion experiments, we provide insights into the specificity of the antibody response following XBB.1.5 vaccination. Although a proportion of vaccinees mounted XBB.1.5-specific antibodies, the magnitude of the response was low. This is consistent with recent work by Liang and colleagues (23, 24), describing that most of the response to the XBB.1.5 vaccine is cross-reactive with only low levels of binding and neutralizing antibodies being detected after Wuhan-1 spike-binding antibody depletion. A large proportion of XBB.1.5-reactive antibodies that cross-react with ancestral SARS-CoV-2 are present due to antigenic relatedness, which is consistent with the imprinting phenomenon in which recall responses toward conserved epitopes seen during the first exposures in life are elicited upon exposure to antigenically distinct viruses (25). For SARS-CoV-2, imprinting has been assessed in humans by directly measuring Ag-specific B cells and monoclonal antibodies (13, 26). The imprinting phenomenon seems to be reproducible in mice, where, for example, groups receiving a primary series of ancestral mRNA vaccine become imprinted to ancestral antigens, as compared to those receiving multiple doses of a BA.1 mRNA vaccine. Moreover, imprinting by ancestral vaccines seems to be partially overcome by multiple exposures to antigens of the Omicron lineage—BA.1 in this case—after primary vaccination with ancestral antigens (23). To further explore the extent of this cross-reactivity, we measured binding antibodies against the spike protein of JN.1, BA.2.87.1 and SARS-CoV-1, and the S2 domain of SARS-CoV-2 spike, as well as neutralizing antibodies against SARS-CoV-1 using a single cycle pseudoviral particle-based entry assay. It was reassuring to see that all the responses measured were efficiently boosted, suggesting that the monovalent XBB.1.5 vaccine induces a robust cross-reactive response.

One unique feature of our cohort is that we included a 3 month time point after the XBB.1.5 vaccination, which allowed us to characterize the durability of the antibody responses. We found that binding and neutralizing antibodies were maintained for at least 3 months, indicating that these cross-reactive responses are durable. In terms of the neutralizing activity against recent circulating variants, the pattern is consistent with recent data from Wang et al. (16). Following the XBB.1.5 vaccination, relative neutralization with respect to WA.1 increased for XBB.1.5 and HV.1 but to a lesser extent for JN.1, which suggests that updating the vaccine for the 2024/2025 Northern hemisphere season to JN.1-derived variant spikes was indeed warranted.

Finally, the lack of IgM antibodies at baseline or after vaccination in most of the vaccinees points to a highly matured antibody response with limited *de novo* responses against the XBB.1.5 spike. The incorporation of updated vaccine antigens of the Omicron lineage in the annually updated SARS-CoV-2 vaccine formulations may redirect some of the antibody response to novel epitopes (14, 27), although a large proportion of the response will be mediated by high levels of cross-reactive antibodies—neutralizing and non-neutralizing—between the ancestral strains and novel SARS-CoV-2 variants. In summary, our data support the notion that subsequent exposures to updated vaccine antigens matching circulating strains will be beneficial to confer enhanced protection.

### Limitations of the study

We described the binding and neutralizing antibody responses up to 3 months after the XBB.1.5 monovalent vaccine administration and provide insights into XBB.1.5-specific antibodies as well as cross-reactivity of these responses. Our study has several limitations. First, the heterogeneity and number of exposures prior to receiving the XBB.1.5 monovalent vaccine, although not significantly reflected in titers at baseline or post-vaccination, may have significant effects on the diversity and specificity of the antibody response. To assess this, it would be necessary to study a larger number of individuals with diverse immune histories and to study the B-cell responses at the monoclonal level. Second, due to the timing of the study, we did not analyze samples collected at later time points post-vaccination (after the 3 month time point), which would be interesting for assessment of long-term durability of these responses. Finally, samples with values below the limit of detection (LoD) in the neutralization assays can affect the accuracy of fold change calculations; i.e., real fold changes can be larger than those estimated. Thus, a high proportion of sera with poor neutralization activity—as seen in this study for the recent Omicron variants or the SARS-CoV-1 pseudovirus—will result in underestimation of the fold difference relative to the pre-vaccine time point. We have indicated this uncertainty by expressing these fold changes as ‘greater than’ the fold change stated (Fig. 1D and F; Fig. 2R).

## MATERIALS AND METHODS

### Experimental model and subject details

Human serum samples were obtained from two different studies: (i) observational longitudinal clinical sample collection from patients with emerging viral infections and (ii) Protection Associated with Rapid Immunity to SARS-CoV-2 (15, 28). The total cohort consisted of *n* = 25 healthy individuals. Samples were taken before and after receiving an XBB.1.5 monovalent vaccine (*n* = 18, Pfizer Comirnaty; *n* = 5, Moderna Spikevax; *n* = 2, Novavax). Baseline samples were collected at 22 ± 15 (1–59) days before vaccination. One month and three month samples were collected at 30 ± 7 (21–49) days and 91 ± 5 (83–112) days post-vaccination, respectively. Six individuals (*n* = 5, Pfizer Comirnaty; *n* = 1, Novavax) had breakthrough infections between the 1 and 3 month sampling time points. More detailed information on the cohort design can be found in Fig. 1A and Fig. S1A; Table S1.

### Method details

#### Cells, viruses, pseudoviruses, and recombinant proteins

##### Cell lines

Vero.E6 African green monkey cells overexpressing transmembrane protease serine 2 (TMPRSS2) cells were cultured at 37°C and 5% CO_2_ using Dulbecco’s modified Eagle medium (DMEM, Thermo Fisher Scientific) supplemented with 10% fetal bovine serum (FBS), 1× non-essential amino acids, 100 U/mL penicillin, 100 µg/mL normocin (InvivoGen), and 3 µg/mL puromycin (InvivoGen). HEK-293T cells (ATCC stands for the American Type Culture Collection, CRL-3216) and HEK 293T-ACE2-TMPRSS2 (BEI Resources, no. NR-55293) were maintained in Dulbecco’s modified Eagle medium (Corning, 10-013-CV) supplemented with 10% heat-inactivated fetal bovine serum (GeminiBio, 100-106), 10% L-glutamine (Corning, 25-005-CI), and 1% penicillin-streptomycin (100 U/ml penicillin, 100 ug/mL streptomycin, g/mL streptomycin, Corning, 30–002-CI) and cultured in a 5% carbon dioxide atmosphere at 37°C.

##### Replication-competent SARS-CoV-2 isolates

The SARS-CoV-2 isolate USA-WA.1/2020 was used as a wild-type/ancestral reference (BEI Resources, NR‐52281). The following viral isolates from the Omicron lineage were provided by the Mount Sinai Pathogen Surveillance Program: hCoV-19/USA/NY-MSHSPSP-PV96109/2023 (JN.1), hCoV-19/USA/NY-MSHSPSP-PV88930/2023 (HV.1), and hCoV-19/USA/NY-MSHSPSP-PV76648/2023 (XBB.1.5).

##### Production and titration of single cycle pseudoviruses

Coronavirus spike pseudotyped luciferase reporter lentiviruses were produced by transfection. The day before transfection, 7 million HEK-293T cells were plated in 10 cm tissue culture plates (Corning, 430167). Transfections were performed using polyethylenimine (Polysciences, no. 23966) (29) with the following plasmids: VRC7568_SARS-CoV-2 S_D614G (VRC_LW, lot U794QFH180-2, a gift from Drs. Nicole Doria-Rose, Lingshu Wang, and Qiong Zhou, Humoral Immunology Core Vaccine Research Center, National Institutes of Health) or pCG_SARS-CoV-1 S (humanized) C-V5 IRES GFP (gift from Dr. Frank Kirchhoff, University of Ulm, Germany), along with pNL Gag Pol (gift from Dr. Paul Bieniasz, Rockefeller University) and pHR’ CMV Luciferase (VRC-5601) (30) at a ratio of 1:16:16. Twenty-four hours post-transfection, the media was replaced, and another 24 h later, the culture supernatants were harvested, filtered through surfactant-free cellulose acetate 0.45 µm syringe filters (Corning, 431220), aliquoted, and stored at −80°C. Virus-like particles were titrated by infecting HEK 293T-ACE2-TMPRSS2 plated in a 96-well black cell culture plate with serially diluting (1:3) pseudo virus-like particles. Luciferase activity was measured 48 h post-infection using the Luciferase Assay System (Promega, E4530) in a VICTOR3 multilabel reader. For the entry inhibition assay, we selected an infectious dose that would yield an approximately 200-fold difference between the pseudovirus-alone control and the background control (uninfected cells).

### Binding and avidity

Recombinant proteins (spike and RBD) were expressed as described elsewhere (12). S2 recombinant protein was purchased from Sino Biological (catalog no. 40590-V08B). ELISAs were used to assess antibody binding. Briefly, Immulon plates (Immulon 4HBX, Thermo Fisher Scientific) were coated with 2 µg/mL of recombinant protein in phosphate-buffered saline (PBS) at 50 µL/well at 4°C overnight. The next day, the plates were washed using 1× PBS supplemented with 0.1% Tween-20 (PBS-T), 225 µL/well, using a BioTek 405 microplate washer. Plates were blocked using 3% skimmed milk protein powder in PBS-T for 1–2 h at room temperature. Following blocking, the 3% milk solution was removed from the plates, and plates were filled with 1% milk in PBS-T solution (diluting solution). Initial dilution of samples was 1:100 with twofold serial dilutions performed across the plates 11 times for a final dilution of 1:204,800 and a 100 µL/well final volume. Plates were incubated with serial serum dilutions for 2 h at room temperature. Following this incubation, they were washed 3× on the plate washer with PBS-T at 225 µL/well. Plates were then incubated for 1 h with a Fab-specific anti-human secondary antibody conjugated to horseradish peroxidase (HRP) at 1:9,000 dilution in diluting solution at 50 µL/well. Following the secondary antibody incubation, the plates were washed 3× with 225 µL/well PBS-T. O-phenylenediamine dihydrochloride (OPD, 100 µL/well) substrate (Sigmafast OPD, Sigma-Aldrich) was added. 3M HCl (50 µL/well) (Thermo Fisher Scientific) was used to quench the reaction after 10 minutes. Optical density (OD) at 490 nm was measured using a Synergy 4 (BioTek) plate reader. Binding data were expressed as AUC, calculated based on the OD values obtained from the different dilutions of sera.

For avidity assessments, Immulon plates (Immulon 4HBX, Thermo Fisher Scientific) were coated and blocked as described above, and sera were diluted in the same manner. Testing entails treatment of plates with urea (chaotropic agent to assess antibody avidity). Binding in urea-treated and non-treated wells was assessed in the same plates to prevent plate-to-plate variations. Following serum incubation, plates were washed 3× on a plate washer with PBS-T 225 µL/well. Urea solution (8 M, 50 µL/well) was added to the treated wells, and PBS (50 µL/well) was added to the untreated wells. Plates were allowed to sit for 10 minutes with the urea or PBS solutions and then washed 3× with 225 µL/well PBS-T. Plates were then incubated with the secondary antibody and developed as described above.

### Microneutralization assays with replication-competent SARS-CoV-2 isolates

Ninety-six-well cell culture plates were coated with 20,000 TMPRSS2 cells per well in DMEM (described above) and incubated overnight at 37°C with 5% CO_2_. Sample dilutions were performed in round-bottom tissue culture 96-well plates using minimum essential medium (1× MEM) supplemented with 0.21% bovine serum albumin (BSA; MP Biomedicals), 100 U/mL penicillin, 100 µg mL^−1^ streptomycin (Gibco), 0.12% sodium bicarbonate, 1% L-glutamine, and 1% 4-(2-hydroxyethyl)-1-piperazineethanesulfonic acid. Initial dilutions of 1:10 were performed, followed by threefold dilutions up to a final dilution of 1:7,290. Diluted serum (80 µL) was transferred from each well of the dilution plate to a corresponding round-bottom “virus plate.” All the following steps were performed in a biosafety level 3 (BSL-3) facility. Viruses were diluted to a concentration of 10,000 50% tissue culture infectious doses/mL in 1× MEM. Diluted virus (80 µL) was added to each well with the diluted sample in the virus plate. Virus plates were incubated with the sera for 1 h at room temperature. After this incubation, cell plates were retrieved from the incubator, and cell culture media was aspirated. The virus-serum mix (120 µL/well) was gently dispensed down the side of the wells of the cell plates. After the inoculum was added to the cell plates, they were returned to the incubator at 37°C with 5% CO_2_ to incubate for an hour. The inoculum was aspirated, and 100 µL of the initial serial dilutions was carefully added to the corresponding wells of the cell. An additional 100 µL/well of 1× MEM with 2% FBS was added to each well for a final volume of 200 µL/well. Cell plates were then incubated for 48 h at 37°C with 5% CO_2_.

Inside the BSL-3 facility, cells were fixed by aspirating the virus-antibody mix and adding 172 µL/well of formaldehyde with 10% methanol for 24–72 h at 4°C. After this inactivation step, nucleoprotein staining was performed. Briefly, cell plates are washed with 200 µL/well of PBS. Following this, the cell membranes were permeabilized by adding 150 µL/well of 0.1% Triton X-100 in PBS for 15 minutes at room temperature. Plates were washed with 200 µL/well of PBS and blocked using 3% BSA (MP Biomedicals) solution in PBS for 1 h at room temperature. One hundred microliters per well of 1 µg/mL biotinylated anti-NP 1C7C7 antibody (mouse anti-SARS NP monoclonal antibody produced at the Icahn School of Medicine at Mount Sinai) diluted in 1% BSA-PBS was added to the plates for 2 h at room temperature. Plates were then washed 2× with 200 µL/well of PBS, and 100 µL/well of HRP-streptavidin (Thermo Fisher Scientific) diluted 1:2,000 in 1% BSA-PBS was added for 1 h at room temperature. Plates were washed 2× with 200 µL/well of PBS, and 100 µL/well of OPD solution (Sigmafast OPD, Sigma-Aldrich) was incubated with the plates for 10 minutes. The reaction was stopped with 50 µL/well of 3M HCl (Thermo Fisher Scientific). OD was measured at 490 nm using a Synergy 4 (BioTek) plate reader. Results were reported as 50% inhibitor dilution (ID_50_). The LoD of the assay is the minimum dilution (1:10) of serum prior to the addition of the virus.

### Single-cycle pseudovirus-based SARS-CoV-1 and SARS-CoV-2 entry assays

Human plasma samples were heat-inactivated at 56°C for 45 minutes and centrifuged at 4,000 × *g* for 10 minutes prior to use in the entry assay. For the luciferase-based entry inhibition assay, 293T-ACE2-TMPRSS2 cells were plated onto poly-L-lysine-coated black cell culture treated 96-well plates (Corning, 3916) a day before infection. Cell density was 2 × 10^4^/well for the SARS-CoV-2 lentivirus VLP assay and 3 × 10^4^/well for the SARS-CoV-1 lentivirus VLP assay. On the day of infection, plasma samples were serially diluted (1:10 ratio followed by six threefold dilution steps) in culture media containing the SARS-CoV-1 or SARS-CoV-2 spike pseudotyped luciferase reporter lentiviruses and incubated for 45 minutes at 37°C with 5% carbon dioxide. Following the incubation step, the mixture of plasma and pseudotyped VLPs was transferred to a 96-well plate seeded with 293T-ACE2-TMPRSS2 target cells. The following controls were included in each plate: serially diluted nevirapine as inhibition positive control, untreated (no plasma and no nevirapine) pseudotyped VLPs as positive control for infection, and uninfected and untreated 293T-ACE2-TMPRSS2 target cells as negative control. Luciferase activity was measured 48 h post-infection using the Luciferase Assay System (Promega, E4530). Luminescence was measured in a VICTOR3 (PerkinElmer) multilabel reader. Prior to testing the full plasma panel, we evaluated the consistency of the entry inhibition assays by testing a subset of 10 plasma specimens with both SARS-CoV-1 and SARS-CoV-2 spike pseudotyped lentivirus VLPs (data not shown). The ID_50_ values calculated for the pseudovirus-based neutralization assays represent the titer (dilution) >50% inhibition midpoint of a variable-slope logistic curve fitted to normalized luminescence data for each serial dilution in Prism version 10 (non-linear least squares fit, log[inhibitor] vs normalized response).

### Serum depletion

Magnetic beads were coupled with SARS-CoV-2 spike or RBD recombinant protein produced in-house, as previously described (12). Briefly, serum was diluted 1:10 in PBS, and 20 µL of spike or RBD coupled to beads was added to the diluted serum and incubated for 2 h at 4°C with constant shaking. Depleted serum (1×) was separated from the magnetic beads using a magnetic stand. Fresh spike (20 µL) or RBD coupled magnetic beads were incubated with the 1× depleted sera for a second incubation overnight at 4°C. Depleted sera (2×) were isolated from the beads using the magnetic stand and were further used for binding assays. Throughout the article, “reactive antibodies” refer to antibodies directed to the spike/RBD of a particular strain measured by ELISA, while the term “specific” is used when referring to antibodies which are directed specifically to the spike/RBD of a particular strain and are not depleted after incubation with antigens of the other strains tested. These specific antibodies are detected by performing depletion experiments prior to ELISA testing.

### Statistical methods

AUC and ID_50_ values were log transformed for statistical analyses. Statistical significance was assessed using a non-parametric analysis of variance (Friedman) test including a post-test for multiple comparisons (corrected Dunn’s test). The correlations were calculated by a non-linear regression using a log-log line – X and Y fit function. All statistical analyses were computed using Prism version 10 software for Mac OS.

## Data Availability

All data are available from ImmPort under the following identifier: SDY2762. No code was used or developed for this study. Any additional information required to reanalyze the data reported in this paper is available upon request. Requests for resources and reagents should be directed to Florian Krammer.

## SUPPLEMENTAL MATERIAL

Key resource table mbio.03607-24-s0001.pdf Key resources.

Figure S1 mbio.03607-24-s0002.tiff Comparison between binding antibodies and virus neutralization titers.

Figure S2 mbio.03607-24-s0003.tiff Immune histories of the study participants.

Legends mbio.03607-24-s0004.docx Legends for Table S1 and Figures S1 and S2.

Table S1 mbio.03607-24-s0005.pdf Demographics and immune history metadata.

ASM does not own the copyrights to Supplemental Material that may be linked to, or accessed through, an article. The authors have granted ASM a non-exclusive, world-wide license to publish the Supplemental Material files. Please contact the corresponding author directly for reuse.
