# Supplementary material for: XBB.1.5 monovalent vaccine induces lasting cross-reactive responses to SARS-CoV-2 variants such as HV.1 and JN.1, as well as SARS-CoV-1, but elicits limited XBB.1.5 specific antibodies
Source: mBio. 2025 Mar 5;16(4):e03607-24. doi: 10.1128/mbio.03607-24 (PMC11980561; doi:10.1128/mbio.03607-24)
Supplement: Key resource table — Key resources. [file mbio.03607-24-s0001.pdf]

Key resources table

Reagent	Source	Identifier
**Antibodies**		
Anti-human IgG (Fab-specific) horseradish peroxidase conjugated antibody	Sigma-Aldrich	Cat# A0293, RRID:AB_257875
Mouse anti-SARS nucleoprotein biotinylated monoclonal antibody (1C7C7)	Center for Therapeutic Antibody Development at the Icahn School of Medicine at Mount Sinai	
**Viruses and Recombinant Proteins**		
SARS-CoV-2 isolate USA-WA1/2020 strain	BEI Resources	NR-52281
SARS-CoV-2 isolate XBB.1.5 (hCoV-19/USA/NY-MSHSPSP-PV76648/2022)	Mount Sinai Pathogen Surveillance Program	
SARS-CoV-2 isolate HV.1 (hCoV-19/USA/NY-MSHSPSP-PV88930/2023)	Mount Sinai Pathogen Surveillance Program	
SARS-CoV-2 isolate JN.1 (hCoV-19/USA/NY-MSHSPSP-PV96109/2023)	Mount Sinai Pathogen Surveillance Program	
6xHis-tagged recombinant SARS-CoV-2 S2 protein	SinoBiological	40590-V08B
6xHis-tagged recombinant SARS-CoV-2 wild type full length spike protein	Krammer Laboratory at Icahn School of Medicine at Mount Sinai	https://labs.icahn.mssm.edu/krammerlab/reagents/
6xHis-tagged recombinant SARS-CoV-2 XBB.1.5 variant full length spike protein	Krammer Laboratory at Icahn School of Medicine at Mount Sinai	https://labs.icahn.mssm.edu/krammerlab/reagents/
6xHis-tagged recombinant SARS-CoV-2 JN.1 variant full length spike protein	Krammer Laboratory at Icahn School of Medicine at Mount Sinai	https://labs.icahn.mssm.edu/krammerlab/reagents/
6xHis-tagged recombinant SARS-CoV-2 wild type RBD protein	Krammer Laboratory at Icahn School of Medicine at Mount Sinai	https://labs.icahn.mssm.edu/krammerlab/reagents/
6xHis-tagged recombinant SARS-CoV-2 XBB.1.5 variant RBD protein	Krammer Laboratory at Icahn School of Medicine at Mount Sinai	https://labs.icahn.mssm.edu/krammerlab/reagents/
**Cells**		
African green monkey Vero-E6 cells expressing Transmembrane protease serine 2 (TMPRSS2)	BPS Biosciences	Cat# 78081
Expi293F cells	Gibco	Cat# A14527

**Software**		
Prism 10	GraphPad	https://ww.graphpad.com
**Biological Samples**		
Human serum samples from the PARIS (Protection Associated with Rapid Immunity to SARS-CoV-2) Study	Simon Laboratory at Icahn School of Medicine at Mount Sinai	https://labs.icahn.mssm.edu/simonlab/
**Chemicals and Reagents**		
SIGMAFAST™ OPD (*o*-phenylenediamine dihydrochloride)	Sigma-Aldrich	Cat# P9187
3M hydrochloric acid	Thermo Fisher Scientific	Cat# S25856
Tween 20	Fisher Bioreagents	Cat# BP337-100
Fat-free Dry Milk	American Bio	Cat# AB10109-01000
