# Supplementary figures and images for: XBB.1.5 monovalent vaccine induces lasting cross-reactive responses to SARS-CoV-2 variants such as HV.1 and JN.1, as well as SARS-CoV-1, but elicits limited XBB.1.5 specific antibodies
Source: mBio. 2025 Mar 5;16(4):e03607-24. doi: 10.1128/mbio.03607-24 (PMC11980561; doi:10.1128/mbio.03607-24)

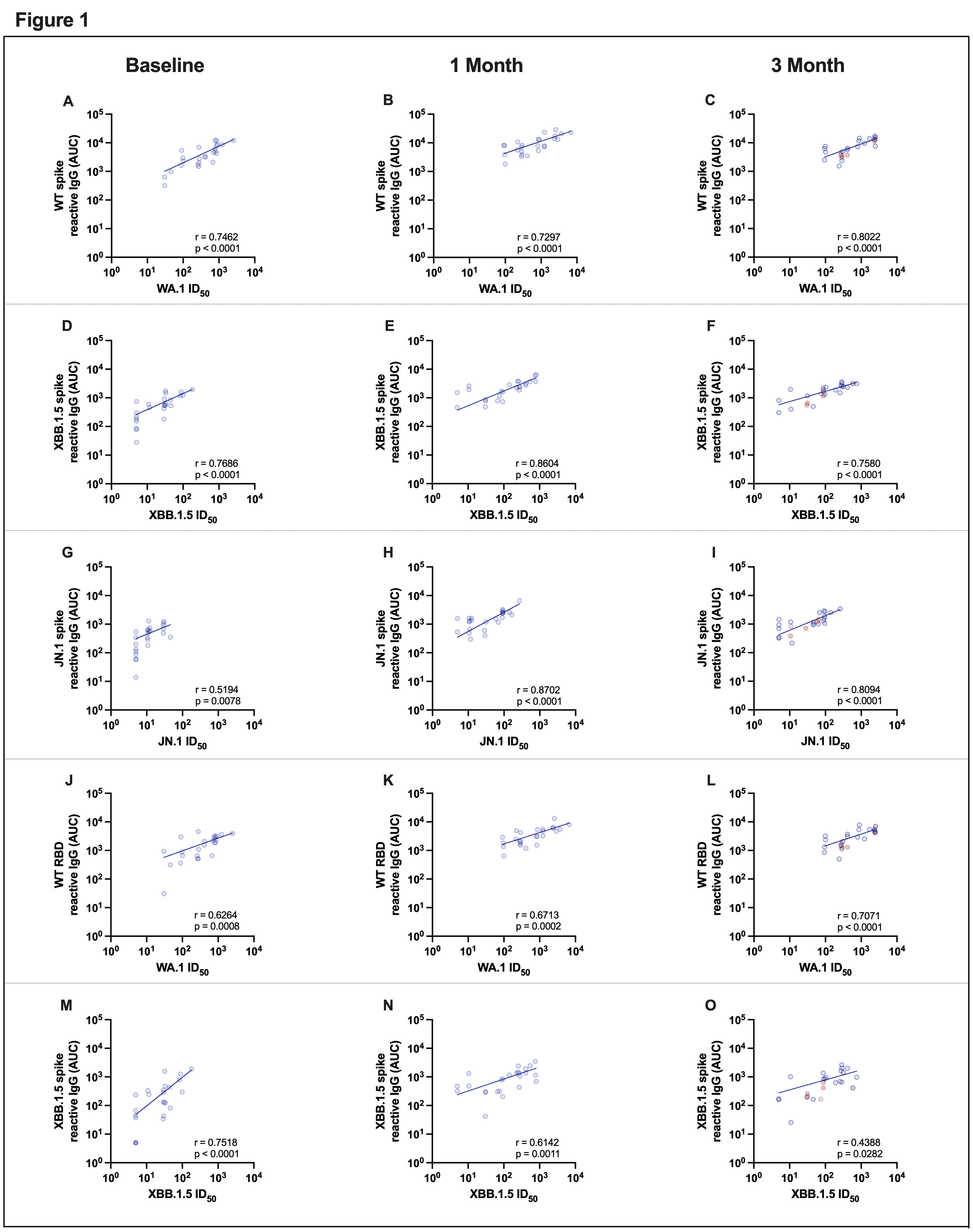

Supplement: Figure S1 — Comparison between binding antibodies and virus neutralization titers. [file mbio.03607-24-s0002.tiff]

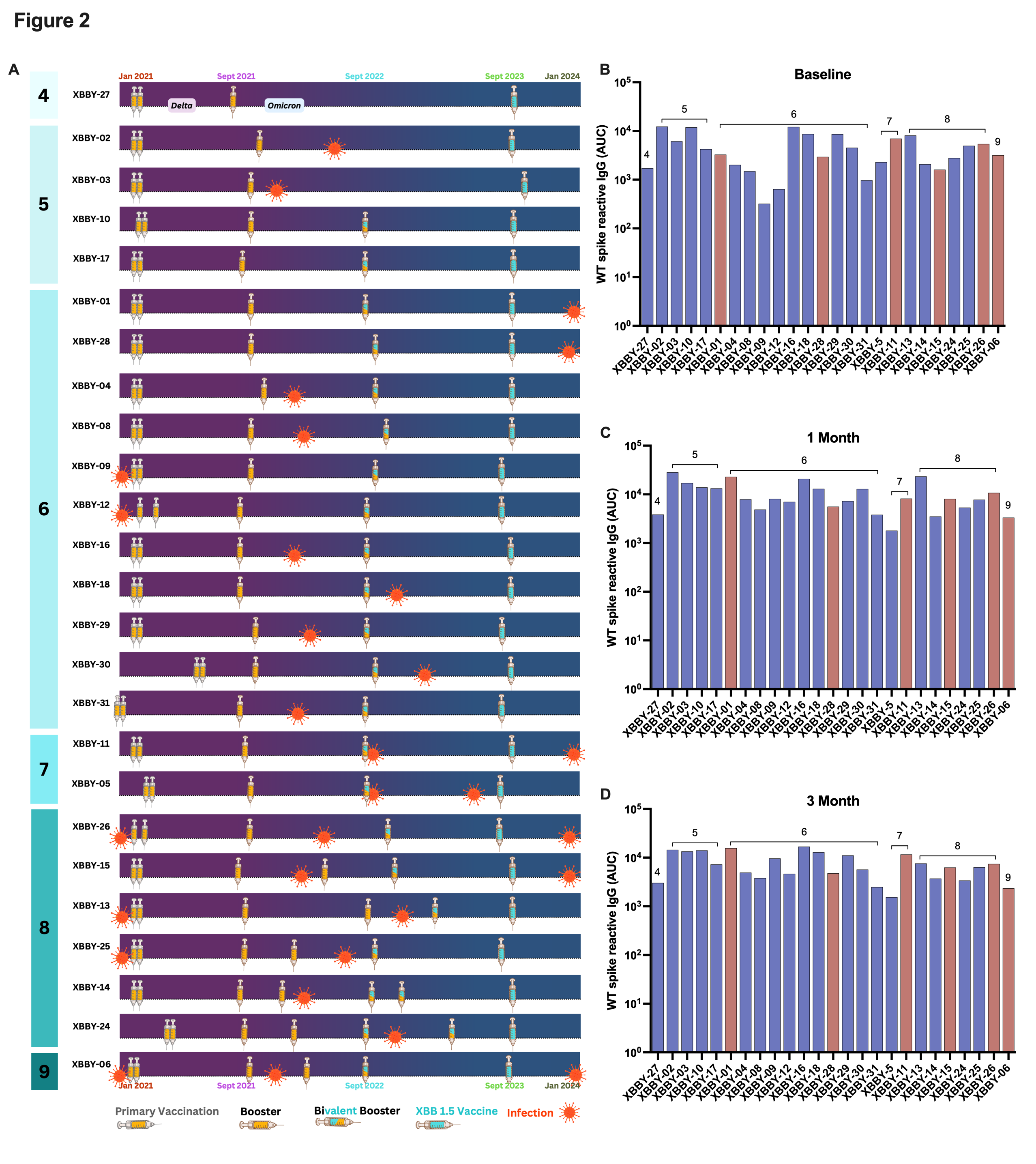

Supplement: Figure S2 — Immune histories of the study participants. [file mbio.03607-24-s0003.tiff]
