# Supplementary material for: XBB.1.5 monovalent vaccine induces lasting cross-reactive responses to SARS-CoV-2 variants such as HV.1 and JN.1, as well as SARS-CoV-1, but elicits limited XBB.1.5 specific antibodies
Source: mBio. 2025 Mar 5;16(4):e03607-24. doi: 10.1128/mbio.03607-24 (PMC11980561; doi:10.1128/mbio.03607-24)
Supplement: Legends — for Table S1 and Figures S1 and S2. [file mbio.03607-24-s0004.docx]

**Supplementary Table 1. Demographics and immune history metadata.**

**Supplementary Figure 1. Correlation of binding to neutralizing antibodies at different time points after the XBB.1.5 monovalent vaccine.** Correlation between binding antibody levels against ancestral, XBB.1.5 or JN.1 spike or against the receptor binding domain (RBD) of ancestral and XBB.1.5 virus, and neutralization against WA.1, XBB.1.5 or JN.1 viruses (**A-E**). Correlations at baseline (left column), 1-month (center column), and 3-months (right column) after the XBB.1.5 monovalent vaccine are shown. AUC: Area Under the Curve. ID_50_: Inhibitory Dilution 50%. Correlation coefficient (r) and significance value (p) are indicated above the x axis. Reactive antibodies: antibodies directed to the spike/RBD of a particular strain.

**Supplementary Figure 2. Association of the magnitude of the antibody response to the spike protein of ancestral SARS-CoV-2 and the number of exposures to infection and vaccination.** Participants were stratified by the number of exposures to SARS-CoV-2 infection or COVID-19 vaccination. Individual exposure histories are indicated in the horizontal bars by the corresponding icons. Number of exposures is indicated by the vertical green bar on the left side (**A**). Antibody levels against the spike protein of ancestral/wild-type (WT) virus in individuals stratified by the number of exposures to infection or vaccination are shown (**B-D**). Antibody levels at baseline (**B**), 1-month (**C**), and 3-months (**D**) after the XBB.1.5 monovalent vaccine are shown. The number of antigenic exposures is indicated on top the bars. AUC: Area Under the Curve. In **B-D**, individuals with a breakthrough infection between 1- and 3- months post-vaccination (n=6) are highlighted in red. Reactive antibodies: antibodies directed to the spike/RBD of a particular strain.
