# Supplementary material for: XBB.1.5 monovalent vaccine induces lasting cross-reactive responses to SARS-CoV-2 variants such as HV.1 and JN.1, as well as SARS-CoV-1, but elicits limited XBB.1.5 specific antibodies
Source: mBio. 2025 Mar 5;16(4):e03607-24. doi: 10.1128/mbio.03607-24 (PMC11980561; doi:10.1128/mbio.03607-24)
Supplement: Table S1 — Demographics and immune history metadata. [file mbio.03607-24-s0005.pdf]

Table S1. Demographics and immune history metadata. Description of the 25 study participants from whom samples collected before and after XBB 1.5 vaccination were analyzed.

**Participant Metadata Summary** (n=25)			
**Demographics**			
Age	Mean	45.0	
	Range	24-74	
		N	*%*
Sex at birth	Male	10	*40%*
	Female	15	*60%*
Race	Caucasian	20	*80%*
	Asian	4	*16%*
	African American	1	*4%*
	Other	0	*0%*
Ethnicity	Hispanic or Latino	1	*4%*
	Not Hispanic or Latino	23	*92%*
	Unknown or not reported	1	*4%*
**SARS-CoV-2 Vaccination and Infection History**			
		N	*%*
XBB 1.5 vaccine type	Pfizer Comirnaty	18	*72%*
	Homogenous	*10*	*40%*
	Heterogenous	*8*	*32%*
	Moderna Spikevax	5	*20%*
	Homogenous	*3*	*12%*
	Heterogenous	*2*	*8%*
	Novavax	2	*8%*
	Homogenous	*0*	*0%*
	Heterogenous	*2*	*8%*
XBB.1.5 vaccine dose number	3	0	*0%*
	4	3	*12%*
	5	16	*64%*
	6	4	*16%*
	7	2	*8%*
Number of infections prior to XBB 1.5 vaccination	0	5	*20%*
	1	15	*60%*
	2	5	*20%*
Infection prior to primary SARS-CoV-2 immunization	Yes	6	*24%*
	No	19	*76%*
One or more breakthrough infections prior to XBB 1.5 vaccination	Yes	20	*80%*
	No	5	*20%*
Breakthrough infection after XBB 1.5 vaccination	Yes	6	*24%*
	No	19	*76%*
Number of total SARS-CoV-2 immune events (vaccination, infection)	4	1	*4%*
	5	4	*16%*
	6	11	*44%*
	7	2	*8%*
	8	6	*24%*
	9	1	*4%*
